# Long-distance dispersal in a fire- and livestock-protected savanna

**DOI:** 10.1002/ece3.515

**Published:** 2013-03-07

**Authors:** Roberto Tarazi, Alexandre M Sebbenn, Paulo Y Kageyama, Roland Vencovsky

**Affiliations:** 1 Departamento de Genética, Universidade de São PauloCP 9, Piracicaba, SP, 13418-900, Brazil; 2 Instituto Florestal de São PauloCP 339, Piracicaba, SP, 13400-970, Brazil; 3 Departamento de Ciências Florestais, Universidade de São PauloCP 9, Piracicaba, SP, 13418-900, Brazil

**Keywords:** *Copaifera*, microsatellite loci, seed dispersal, pollen dispersal, parentage analysis, spatial genetic structure

## Abstract

Savannas are highly diverse and dynamic environments that can shift to forest formations due to protection policies. Long-distance dispersal may shape the genetic structure of these new closed forest formations. We analyzed eight microsatellite loci using a single-time approach to understand contemporary pollen and effective seed dispersal of the tropical tree, *Copaifera langsdorffii* Desf. (Fabaceae), occurring in a Brazilian fire- and livestock-protected savanna. We sampled all adult trees found within a 10.24 ha permanent plot, young trees within a subplot of 1.44 ha and open-pollinated seeds. We detected a very high level of genetic diversity among the three generations in the studied plot. Parentage analysis revealed high pollen immigration rate (0.64) and a mean contemporary pollen dispersal distance of 74 m. In addition, half-sib production was 1.8 times higher than full-sibs in significant higher distances, indicating foraging activity preference for different trees at long distances. There was a significant and negative correlation between diameter at breast height (DBH) of the pollen donor with the number of seeds (*r* = −0.640, *P*-value = 0.032), suggesting that pollen donor trees with a higher DBH produce less seeds. The mean distance of realized seed dispersal (recruitment kernel) was 135 m due to the large home range dispersers (birds and mammals) in the area. The small magnitude of spatial genetic structure found in young trees may be a consequence of overlapping seed shadows and increased tree density. Our results show the positive side of closed canopy expansion, where animal activities regarding pollination and seed dispersal are extremely high.

## Introduction

The Brazilian savanna, known as “cerrado”, covers an area of 2,000,000 km^2^ from 3°N to 24°S and represents approximately 23% of the country's land surface (Klink and Machado 2005). The cerrado consists of a mosaic of physiognomies that vary along a gradient from open grasslands to dense woodland formations controlled by interactions among climatic, edaphic, and disturbance factors such as fire and herbivory (Roitman et al. 2008). Human activities has destroyed and fragmented more than 55% of the Brazilian cerrado (Klink and Machado 2005). Efforts to protect this domain in conservation units may shift the ecosystem dynamics and plant species evolution, because of fire-protection policy (Klink and Machado 2005; Roitman et al. 2008; Pinheiro and Durigan 2009). Long-term fire-suppression allows expansion and establishment of forest systems over other savanna formations, as reported in Australia (Russell-Smith et al. 2004; Tng et al. 2012), Brazil (Felfini et al. 2000; Moreira 2000; Henriques and Hay 2002; Roitman et al. 2008; Pinheiro and Durigan 2009), India (Puyravaud et al. 1994), and South Africa (Higgins et al. 2007). The forest expansion comes with the increase in tree density, dominance of canopy-forming species (Roitman et al. 2008; Pinheiro and Durigan 2009; Tng et al. 2012; Buitenwerf et al. 2012), and high immigration rates (Roitman et al. 2008). Understanding dispersal patterns in the dynamics of expanding populations may help predict the genetic fate of plant populations in fragmented habitats (Troupin et al. 2006; Chung et al. 2011; Steinitz et al. 2011).

Plant colonization events rely on the interaction with abiotic factors and animals to realize successful pollen and seed dispersal, which are two independent and critical aspects of gene flow (Nathan 2006; Dick et al. 2008; Muller-Landau et al. 2008). Dispersal is the main factor in determining levels of spatial genetic structure (SGS) seen within expanding plant populations (Hamrick et al. 1993; Epperson 2003; Chung et al. 2011; Choo et al. 2012). SGS is a nonrandom distribution of genotypes that is predominately a consequence of limited pollen and seed dispersal (Epperson 2003; Vekemans and Hardy 2004). Pollen dispersal patterns are dependent on the population's demographic structure. Generally, high population density and clumped distribution tend to lower pollen dispersal distances (Ward et al. 2005; Robledo-Arnuncio and Austerlitz 2006). Combined to low reproductive population size, the magnitude of SGS may increase even before seed dispersal (Hardy et al. 2006; Tarazi et al. 2010a; Sebbenn et al. 2011). Moreover, long-distance seed dispersal and establishment from fleshy fruits is dependent on its relationship with the frugivorous community and negative density- and distance-dependent mechanisms (Clark and Clark 1984; Nathan and Casagrandi 2004; Garcia and Grivet 2011). The Janzen-Connell (JC) escape hypothesis predicts a hump-shaped recruitment pattern as a result of the dispersal kernel with an increase in survival probability with increasing distance from the conspecific parent (Janzen 1970; Connell 1971; Steinitz et al. 2011). Consequently, density-dependent mechanisms that result in thinning and distance-dependent mechanisms, which enhance gene dispersal may reduce the magnitude of SGS in older age classes (Born et al. 2008; Chung et al. 2011). Because SGS might arise in spite of frequent kin-structured long-distance seed dispersal (Torimaru et al. 2007), many studies using genetic markers demonstrate the existence of SGS even in older age classes (Vekemans and Hardy 2004; Hardy et al. 2006; Dick et al. 2008; Tarazi et al. 2010a,b; Sebbenn et al. 2011).

Genetic markers, such as microsatellite (SSR) loci, provide means to resolve long-standing questions in dispersal ecology of tree species (Ashley 2010). With animal-dispersed tree species, direct dispersal measures should be preferred over inverse modeling methods, which underestimate the frequency of long-distance dispersal events (Hardesty et al. 2006). For this study, we used SSR markers and a single-time approach to understand contemporary pollen and effective seed dispersal of the tropical tree, *Copaifera langsdorffii* Desf. (Fabaceae), occurring in a fire- and livestock-protected savanna. Previous data obtained in a small fragment (<250 ha) in the Brazilian Atlantic forest (BAF), reveal that this gravity- and animal-dispersed species has a high yield (8368 fruits tree^−1^) supra-annual fruit production. Through direct animal interaction observation, the dispersal estimates were short (14 m) and seed deposition was concentrated mainly underneath the edge of the crown, without asymmetric and leptokurtic distribution. The population diametric distribution presented itself as a reverse J-shape attributed to density-dependent mechanisms (Pedroni 1993). Moreover, genetic studies in an isolated 4.8 ha fragment in the BAF revealed short pollen (<150 m) and seed dispersal (<100 m) distances. Significant values of SGS were found up to 50 m for adult trees and 20 m for seedlings (Sebbenn et al. 2011). *Copaifera langsdorffii* phenotypic plasticity makes it a light and shade tolerant species (Carvalho 2003). Unlike previous studies in the BAF, we address the following facets of *C. langsdorffii* genetic structure and gene dispersal in a permanent plot located in a large (>1000 ha) savanna fragment: (1) the genetic diversity and effective size in adults, young trees, and open-pollinated seeds; (2) the distances seeds disperse and successfully recruit as young trees; (3) the frequency of long-distance (>100 m) recruitment and pollen dispersal; and (4) the relative contribution of seeds and pollen to genetic structure.

Our sets of predictions regarding gene dispersal and genetic diversity patterns in the recently expanded *C. langsdorffii* population are that:

1. The population may exhibit an increase in seed dispersal distance from the parent tree and seed shadow overlap due to density- and distance-dependent mechanisms hence, SGS strength would be weak, even in newly recruited individuals.
2. In this expanded high-density population, we should expect for the majority of the observations that pollen dispersal distances will rarely exceed 150 m and that the largest trees will create reproductive dominance.
3. In this population, density-dependent effects and gene flow would act together to maintain high levels of genetic diversity.

## Materials and Methods

### Study species

**Figure 1:**
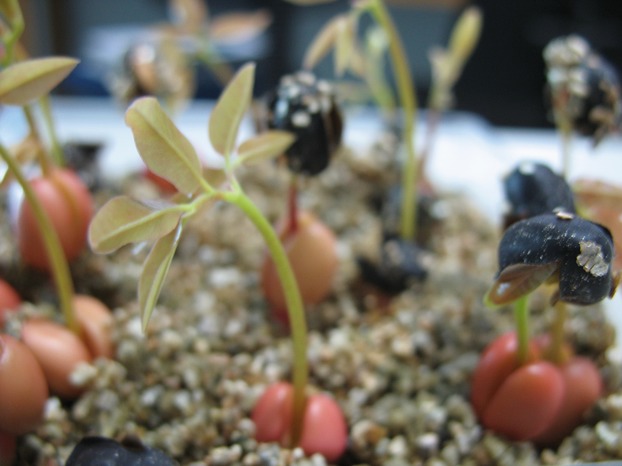
*Copaifera langsdorffii* Desf. (Fabaceae) seedlings.

*Copaifera langsdorffii* Desf. (Fabaceae) is a hermaphroditic tree with wide distribution in the Brazilian savanna and Atlantic rain forest (Carvalho 2003; Fig. 1). The species has a predominant outcrossing mating system with up to 8% of selfing (Sebbenn et al. 2011). Its herkogamic and bisexual flowers are pollinated mainly by small native bees, such as *Scaptotrigona* and *Trigona,* and the invasive bee species *Apis mellifera* (Freitas and Oliveira 2002). In natural populations the reproductive stage starts between 20 and 30 years and the species is known to have a medium- to slow-growth rate (Carvalho 2003). *Copaifera langsdorffii* produces elliptic seeds (average, 14 ×9.6 mm) that weigh approximately 0.9 g surrounded by an abundant colored aril. Ants (*Atta* sp. and *Acromyrmex* sp.), primates (*Brachyteles arachnoides*, *Cebus apella, and Alouatta fusca*), and birds (*Cyanocorax cristatellus*, *Mimus saturninus*, *Ramphastos toco*, *Thraupis sayaca*, *Dacnis cayana Turdus rufiventris*, *Pitangus sulphurantus*, *Tyrannus melancholicus*) are responsible for primary and secondary dispersal events (Pedroni 1993). *Copaifera langsdorffii* is an economically valuable species due to its high quality wood and the oil extracted from its trunk, which has pharmaceutical properties. The species is listed as at risk of extinction in some Brazilian states (Carvalho 2003) due to the destruction of its natural environments, such as the Atlantic rain forest which has been reduced by 84% of the original area (Ribeiro et al. 2009), and savanna, which has been reduced by 35% (Klink and Machado 2005).

### Study site and sampling

**Figure 2:**
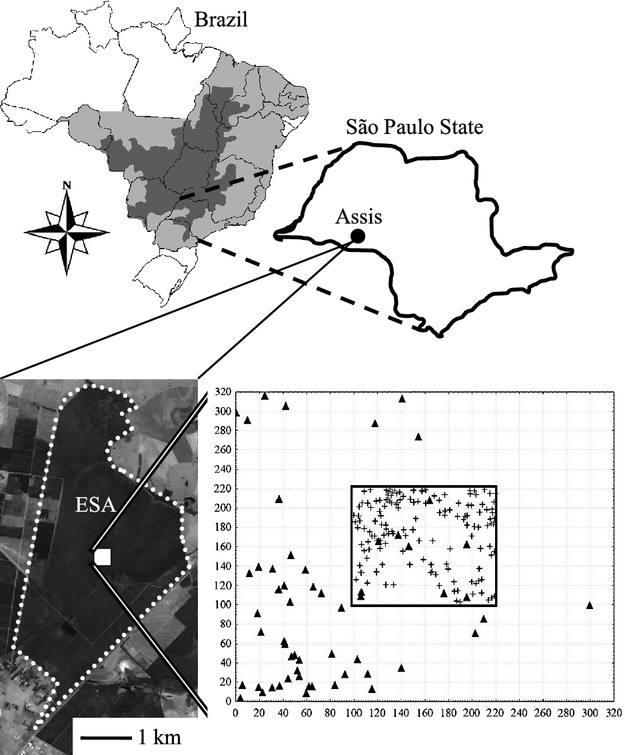
Geographic distribution of *C. langsdorffii* (light gray) and the savanna in Brazil (dark gray). Location of the city of Assis in the State of São Paulo and the 10.24 ha plot (white square) inside the boundaries of the ESA (white dots). Distribution of the 57 adults (▲) throughout the 10.24 ha plot and the 147 young trees (+) in the 1.44 ha subplot in the center of the plot.

This study was conducted in the Ecological Station of Assis (ESA), a reserve in the State of São Paulo, Brazil (22º33′20″ to 22º37′41″S and 50º24′4″ to 50º21′27″W), covering an area of 1,760.64 ha (Fig. 2). Due to the protection of the ESA from fire and livestock grazing beginning in 1962, the closed savanna woodland habitat has increased its area from 53.4 to 91.4% at the expense of other savanna types. This expansion has favored species such as *C. langsdorffii* that is the dominant species in the ESA (Pinheiro and Durigan 2009). The ESA is one of the few protected savanna fragments left in the State of São Paulo and presents a high diversity of bird species, terrestrial small to large size mammals and no presence of primates.

**Figure 3:**
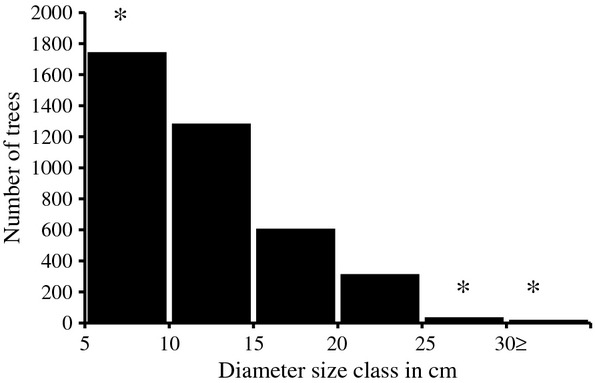
Diameter size class distribution of *C. langsdorffii* in a 10.24 ha plot in a recent expanded woodland savanna. * indicates the selected size classes in the study.

We focused our study in a pre-existing 10.24 ha (320 × 320 m) permanent plot of closed savanna woodland inside the ESA, where a census of all tree species with a diameter at breast height (DBH) higher than 5 cm was completed in 2006 by BIOTA-FAPESP Permanent Plot Consortium. A total of 4009 individuals of *C. langsdorffii* were identified, measured and mapped by the Consortium. For a cost-effective sampling strategy to attain our objectives, we first analyzed *C. langsdorffii* demographic data in the 10.24 ha permanent plot and selected the two extreme sampled size classes (young trees with DBH between 5 and 10 cm and reproductive trees DBH ≥ 25 cm). We based our size class selection on the assumption that the high rates of thinning from one size class to another (Fig. 3) was caused by negative density- and distance-dependent mechanisms. Therefore, we could retrieve different genetic signatures from the two extreme size classes. To investigate genetic diversity and gene dispersal patterns we sampled all adult *C. langsdorffii* trees with DBH ≥ 25 cm (DBH mean of 30.6 cm; height mean of 12.7 m) in the 10.24 ha plot (*N* of 57; density (*d*) of 5.8 trees ha^−1^). To study contemporary gene flow, all adult *C. langsdorffii* that produced flowers and fruits in the plot in 2007 (*N* of 17; *d* of 1.66 trees ha^−1^) had their open-pollinated seeds collected directly from the crown. Twenty random seeds per seed tree were used in our analysis, totaling 340 seeds.

We sampled all young trees with DBH between 5 and 10 cm in a subplot of 1.44 ha (120 × 120 m; *N* of 147; *d* of 102.1 trees ha^−1^; DBH mean of 7.4 cm; height mean of 6.6 m) in the center of the plot because of their high sample size (*N* = 1745) through the 10.24 ha plot (Fig. 2). We can draw three facts through this sampling scheme: (1) we reduced the sample size of young trees to less than 10% of its original size, reducing genotyping costs; (2) we were able to continue sampling of all young trees in a smaller area following Cavers et al. (2005) optimal SGS sampling strategies; (3) with the position of the subplot in the center of plot, we created a 100 m margin to detect long-distance seed dispersal.

Regarding the woodland savanna expansion based on Pinheiro and Durigan (2009) published maps and E.S. Pinheiro (unpubl. data), the study plot in 1962 had approximately 50% of dense savanna, 30% of typical savanna, and 20% of woodland savanna. Our subplot had 50% of dense savanna and 50% of typical savanna. In 1992 the plot had around 90% of woodland savanna and our subplot had 50% of dense savanna and 50% woodland of savanna. In 2006, woodland savanna covered 100% of the plot and subplot. The typical savanna “cerrado *sensu stricto*” is characterized by open grasslands, shrubs and up to 50% of discontinuous tree cover. On the other hand, savanna woodland (SW) “cerradão”, has a forest physiognomy, where the woody vegetation forms a continuous stratum, typically with more than 90% coverage, and the average height of the trees varies between 8 and 15 m (Pinheiro and Durigan 2009). The adult trees of *C. langsdorffii* already existed in 1962 and the majority of the individuals were in the woodland savanna. The young trees in the subplot are result of recent woodland expansion.

### Spatial distribution analysis

To assess the spatial distribution of adult trees in the plot and young trees in the subplot, we used Wiegand and Moloney (2004) univariate O-ring statistic O(*r*). Because the O-ring statistic is annulus-based and noncumulative, it permits direct comparison on the same spatial scale of physical clustering with spatial genetic structure. We tested the significance of O(*r*) for each annulus of radius (*r*) around each tree using the common null model of complete spatial randomness (CSR). The starting ring width was 20 m with a 1 m lag. The first order intensity, λ, was calculated as reference to the point pattern expected under CSR. For each generation, 95% confidence intervals (*CI*_95_) around CSR for a given *r* were constructed from 2.5 and 97.5 percentiles after ranking O(*r*) from 1000 spatial randomizations. An observed value of O(*r*) above *CI*_95_ indicates significant spatial clustering, within *CI*_95_ spatial randomness and below *CI*_95_ spatial repulsion (hyper-dispersion), at radius *r*. All calculations and simulations were conducted using the program PROGRAMITA (Wiegand 2003).

### Microsatellite genotyping

DNA was extracted from the leaves of adult and young trees and from leaflets of germinated seeds using the CTAB method (Doyle and Doyle 1990). After extraction, DNA was quantified on 1% agarose gels with a lambda DNA standard and diluted to a concentration of 2.5 ng/μL. The eight dinucleotide microsatellite loci used in this study and the PCR conditions were reported by Ciampi et al. (2000) and Tarazi et al. (2010b). The amplifications were performed using an MJ Research PT-100 thermal cycler (MJ Research, Matertown, MA, USA) and amplification products were separated on 5% (w/v) polyacrylamide gels stained with silver nitrate. As microsatellite genotyping errors can lead to biased results (Hoffman and Amos 2005), we used control genotypes and double-checked all gels. We used MICRO-CHECKER 2.2.3 (Oosterhout et al. 2004) to test possible stuttering, large allelic dropout, and null alleles in the adult individuals, using a 95% confidence interval based on 1000 Monte-Carlo permutations. No evidence for scoring errors due to stuttering or for large allelic dropout was found. Null alleles may be present at loci CL02, CL32, and CL34, as is suggested by the general excess of homozygotes for most allele size classes (*P*-value < 0.05). However, loci CL02 and CL34 had a mistyping of zero, and locus CL32 had only one mistyping, indicating that the homozygote excess may be due to selfing in the population and not necessarily to null alleles. Another method that may demonstrate the absence of null alleles is the check for genotyping errors and null alleles that we performed using MLTR 3.4 (Ritland 2002) to detect seed tree-offspring mismatches. We obtained zero seed tree-offspring mismatching and an absence of null alleles in the offspring for all loci.

### Genetic diversity and fixation index analysis

The genetic diversity of the three generations (adult, young trees, and seeds) was estimated by the number of alleles per locus (*k*), allelic richness (*k*_R_), observed heterozygosity (*H*_o_), and expected heterozygosity at Hardy-Weinberg equilibrium (*H*_e_). The level of inbreeding for each generation was estimated using the fixation index (*F*). To verify the significance of *F* values, we applied 10,000 permutations for all loci and sequential Bonferroni correction for multiple comparisons (α = 0.05). We calculated a *CI*_95_ using Jackknife procedure over all loci for comparison of the averages of *k*, *k*_R_, *H*_o_, *H*_e_, and *F* among generations. All estimates were calculated using the FSTAT program (Goudet 1995).

### Estimate of the effective population size

The effective population size (*N*_e_) was estimated for adult and young trees following Cockerham (1969): *N*_e_ = 0.5/*Θ*, where *Θ* is average coancestry coefficient of the generation under consideration. The average coancestry coefficient among generations was estimated using the expression: 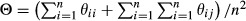, where *n* is the sample size, *θ*_*ii*_ is the estimate of self-coancestry [*θ*_*ii*_ = 0.5(1 + *F*_*i*_), where *F*_*i*_ is the inbreeding coefficient for individual *i*], and 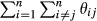 is the sum of all estimates of coancestry between pairs of individuals in a population, excluding self-coancestry.

To detect recent reductions in effective population size due to thinning in adult and young trees we used the Wilcoxon significance test with 5000 iterations under Stepwise Mutation Model (SMM) (Ohta and Kimura 1973) and Two-phased Mutation Model (TPM) (Di Rienzo et al. 1994) implemented in BOTTLENECK (Piry et al. 1999). SMM and TPM are more suitable for SSR loci, so is the Wilcoxon significance test when less than 20 SSR loci are applied (Luikart and Cornuet 1998). BOTTLENECK tests if the expected heterozygosity calculated from allele frequencies is higher than the heterozygosity expected from the number of alleles in the population assuming mutation-drift equilibrium under SMM and TPM. Positive values of BOTTLENECK statistics reflect a gene diversity excess possibly caused by recent founder events, whereas negative values are consistent with heterozygote advantage.

### Fine-scale spatial genetic structure analysis (SGS)

For the analysis of SGS among adults in the plot and young trees in the subplot we used the *J Nason* coancestry (kinship) coefficient (*θ*_*xy*_), described in Loiselle et al. (1995) and implemented in SPAGEDI 1.3 (Hardy and Vekemans 2002). The parameter *θ*_*xy*_ was calculated for all pairs of individuals and averaged over a set of distance classes and then plotted against these distance. For both adults and young trees, we used seven distance classes (20, 40, 60, 80, 100, 120, and 140 m). To test if there was significant deviation from random structure, the 95% confidence intervals were estimated from 10,000 permutations of the genotypes among the different distance classes. To compare the intensity of SGS of adult and young trees, the *S*_p_ statistic (Vekemans and Hardy 2004) was calculated as 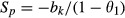, where *θ*_1_ is the average pairwise coancestry coefficient calculated between all individuals within the first distance class (20 m) and *b*_k_ is the slope of the regression of coancestry coefficient on the logarithm of spatial distance separating individual (ln (*d*_*xy*_)). To test for SGS, spatial positions of the individuals were permutated 10,000 times to obtain the frequency distribution of *b*_k_ under the null hypothesis that *θ*_1_ and ln (*d*_*xy*_) were uncorrelated. The coefficients of determination were compared for the linear (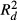) and logarithmic regression (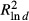) to assess whether SGS of adult and young trees matched predictions of isolation by distance in two dimensions (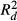
<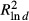) or predictions of contact between two spatially segregated differentiated gene pools, 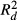
>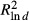 (Rousset 2000). We did not estimate the neighborhood size (*Nb*) because this recently expanded population may not be in equilibrium.

### Parentage analysis

Parentage analysis was carried out by maximum-likelihood maternity and paternity assignment (Meagher 1986) based on multilocus genotyping of 147 young trees from the subplot, 340 open-pollinated seeds and all 57 adult trees from the study plot, using CERVUS 3.0 program (Marshall et al. 1998; Kalinowski et al. 2007). We determined the most likely parents (for seeds) and parent pairs (for young trees) by the Δ statistic (Marshall et al. 1998), using the allele frequencies calculated in the adult population, as suggested by Meagher and Thompson (1986). For young trees we used simulations to determine the two most likely parents (mother and fathers). For seeds, these simulations were performed to determine the most likely father. All 57 adult trees were used as candidate parents (mother and/or father); therefore, selfing (*s*) was considered in the analysis. The critical value of Δ was simulated using a confidence interval of 95% with 100,000 replications, a genotyping error rate of 0.01 per locus and 95% of candidates sampled. True parenthood was confirmed if a candidate individual or pair of candidate individuals had a calculated Δ index higher than the cryptic Δ derived from simulations. The theoretical power to exclude the first parent (*P*_first-parent_) and parent pair (*P*_parent-pair_) was also calculated using CERVUS 3.0. The cryptic gene flow, or probability of assigning a parent candidate inside the population when the true one is outside the population, was calculated as:*P*_m_ = (1 − *P*_first-parent_^*n*^), where *n* is the number of candidates (Dow and Ashley 1996). The estimate of the contemporary outcrossing rate (*t*_seeds_ = 1 − *s*_seeds_) was calculated as the number of outcrossed seeds divided by the total number of assigned seeds in the plot. The pollen immigration rate *m*_pollen_ in seeds was calculated by the proportion of seeds that had no pollen parent inside the plot and subplot in relation to the total number of sampled seeds in each area. Contemporary pollen dispersal distance was based on results from seeds.

The average effective neighborhood area of pollination (*A*_ep_ = 2*πσ*^2^) was calculated from the pollen dispersal variance (*σ*^2^) of each seed tree, assuming a central circular area around it (Levin 1988). The axial pollen dispersal variance (*σ*^2^) was estimated from the distance between seed trees and their pollen donors as identified by paternity analysis. Using Spearman′s correlation coefficient (Sokal and Rohlf 1989), we also performed correlations between spatial distance, *θ*_*xy*_ and DBH with the number of descendants of each pollen donor. In order to investigate if mating success was a function of distance between trees, we compared the frequency distribution of pollen dispersal distance with the frequency distribution of the distance between the adults using a Kolmogorov–Smirnov test (D) (Sokal and Rohlf 1989).

The effective seed immigration rate (*m*_seed_) for young trees within the subplot was calculated by the ratio of young trees with un-assigned parents (mother and father) in the plot to the total number of young trees sampled in the subplot. To calculate seed dispersal distance from the seed tree, based on the results of positive young tree assignment, we used a conservative seed dispersal approach for hermaphroditic species, which the closest parent is assumed to be the maternal parent. First, we recovered the distances of the young trees produced by selfing. Then we added the closest assigned parent as the mother. When we found both parents inside the plot, we were able to estimate the effective seed dispersal distance for full- and half-sibs. In order to investigate if seed dispersal fit a normal or leptokurtic distribution, we compared the frequency distribution of realized seed dispersal distance with the frequency expected for normal or leptokurtic distribution using a Kolmogorov-Smirnov test (D) (Sokal and Rohlf 1989). In this study, when we refer to effective seed dispersal using young trees, we are characterizing the recruitment kernel that combines the net effects of dispersal and post-dispersal survival, with establishment of young trees.

## Results

### Spatial distribution

**Figure 4:**
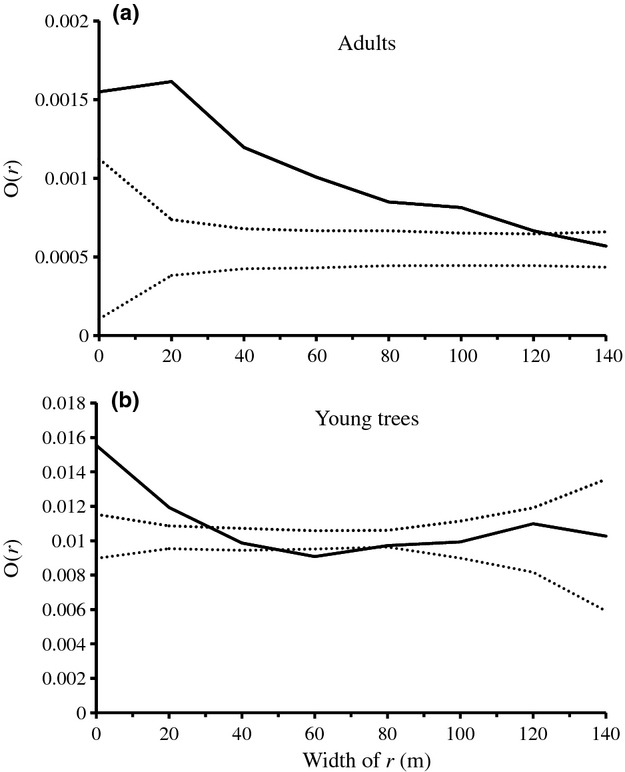
Spatial structures for (a) seven distance classes of adult and (b) young trees of *C. langsdorffii*. The solid line represents the mean O(*r*) and dashed lines 95% confidence interval for an annulus of radius *r* with 1 m lags.

O-ring analyses revealed significant spatial aggregation of adult trees in the plot at *r* = 0–120 m, and young trees in the subplot at *r* = 0–26 m (Fig. 4). The mean distance between the closest neighbors (*R*_o_) was 16.8 m for adult trees and 5.8 m for young trees.

### Genetic diversity and fixation index

We detected a very high level of genetic diversity in the studied plot (Table 1). The number of alleles (*k*) was similar among adults, young trees and seeds. However, allelic richness (*k*_R_) was significantly different among generations. We found eight private alleles in the seeds and six alleles that were lost in this generation in comparison to adults and young trees. Adult trees had higher observed heterozygosity (*H*_o_) than young trees and seeds. For all generations, expected heterozygosity (*H*_e_) was higher than that observed, indicating a deficiency of heterozygotes. The fixation index (*F*) was significantly higher in young trees than in adult trees and seeds (Table 1).

**Table 1:** Genetic diversity and fixation index in adult trees, young trees, and seeds of *C. langsdorffii* at the Ecological Station of Assis

Locus	Adult trees (*n* = 57)					Young trees (*n* = 147)					Seeds (*n* = 340)				
		
	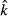	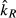	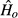	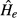	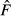	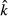	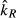	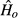	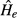	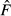	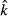	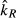	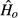	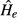	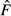
CL01	10	10	0.754	0.795	0.052	10	10	0.745	0.837	0.110	12	11	0.770	0.825	0.067
CL02	16	16	0.789	0.888	0.111	16	16	0.648	0.899	0.280	18	15	0.667	0.877	0.240
CL06	16	16	0.982	0.900	−0.091	17	17	0.966	0.908	−0.064	17	15	0.920	0.890	−0.035
CL20	22	22	0.842	0.916	0.081	22	20	0.822	0.886	0.072	22	20	0.838	0.905	0.075
CL27	13	13	0.891	0.862	−0.034	13	12	0.683	0.881	0.226	13	11	0.847	0.889	0.047
CL32	16	16	0.691	0.904	0.236	19	17	0.801	0.900	0.110	21	14	0.808	0.820	0.014
CL34	18	18	0.684	0.875	0.218	16	14	0.597	0.819	0.272	17	13	0.599	0.795	0.246
CL39	19	19	0.875	0.870	−0.006	19	16	0.753	0.815	0.095	16	15	0.871	0.875	0.004
Mean	16.3	16.3	0.814	0.876	0.071*	16.5	15.3	0.752	0.870	0.138*	17	14.25	0.780	0.849	0.081*
*CI*_95(inf)_	15.9	15.9	0.808	0.873	0.060	16.1	15.14	0.741	0.867	0.134	16.7	14.15	0.783	0.858	0.040
*CI*_95(sup)_	16.7	16.7	0.820	0.879	0.082	16.9	15.36	0.763	0.873	0.142	17.3	14.35	0.797	0.862	0.098
Total	130	130	–	–	–	132	122	–	–	–	136	114	–	–	–

*k* is the number of alleles; *k*_R_ is the allelic richness based on minimum sample size of 55 trees; *H*_o_ is the observed heterozygosity; *H*_e_ is the expected heterozygosity in Hardy–Weinberg Equilibrium; *F* is the fixation index; *CI*_95_ is the 95% confidence interval calculated from a Jackknife procedure among loci; * is the *P*-value < 0.00625.

### Effective population size

The 57 adult trees correspond to a *N*_e_ of 21 and the 147 young trees to a *N*_e_ of 23. The difference between *N* and *N*_e_ is associated with the positive *F* values and to the large number of pairs of individuals with a coancestry coefficient (*θ*_*xy*_) higher than 0.0625 (equivalent to first-degree cousins): 45% in adult and young trees. We detected a significant deficiency of expected heterozygosity at mutation-drift equilibrium under SMM (*P*-value = 0.002) and TPM (*P*-value = 0.019) for adults. On the other hand, young trees where at mutation-drift equilibrium under SMM (*P*-value = 0.170) and TPM (*P*-value = 0.320).

### Fine-scale spatial genetic structure (SGS)

**Figure 5:**
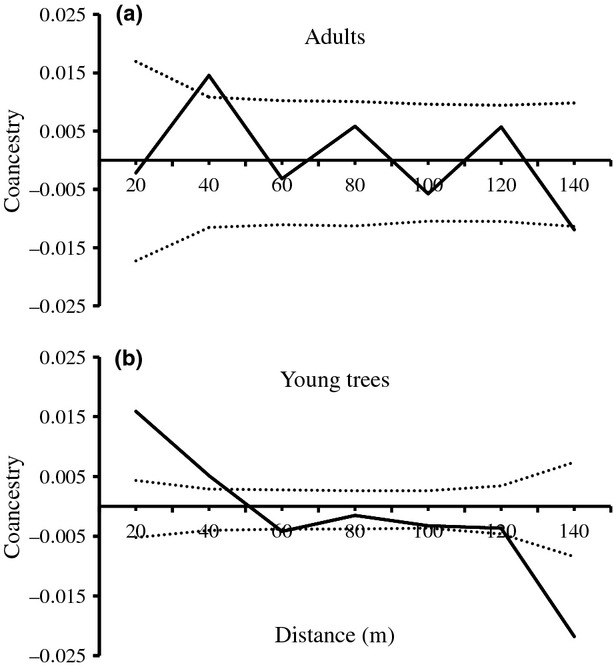
Correlograms of average coancestry coefficient (*θ*_*xy*_) for (a) seven distance classes of adult and (b) young trees of *C. langsdorffii*. The solid line represents the average values and the dashed lines represent the 95% (two-tailed) confidence interval of the average *θ*_*xy*_ distribution.

Our analysis revealed weak but significant SGS among young trees (*θ*_1_ = 0.016; *P*-value < 0.001) up to 40 m and a significant value at 40 m for adults, indicating the formation of family structures (Fig. 5). The intensity of SGS in adult trees (*S*_p_ = 0.0033; *CI*_95_ = −0.0034 to 0.0039) was similar to young trees (*S*_p_ = 0.0089; *CI*_95_ = −0.0020 to 0.0022; *t*-test = 1.86; *P*-value = 0.08). The regression slope of coancestry coefficient on the logarithm of spatial distance (0–140 m) was not significant in adult trees (*b*_k_ = −0.0034; *P*-value = 0.09), but significantly negative in young trees (*b*_k_ = −0.0088; *P*-value < 0.01), confirming isolation by distance (IBD). The coefficients of determination also supported IBD in young trees with 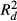
<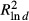 (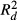 = 0.0045; *CI*_95_ = 0.0044 to 0.0046 and 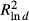 = 0.0054; *CI*_95_ = 0.0053 to 0.0055).

### Parentage analysis

The high polymorphism found in this population of *C. langsdorffii* is reflected in a high exclusion probability of the first parent (*P*_first-parent_ = 0.9999) and parent pair (*P*_parent-pair_ = 0.9999), which demonstrates the effectiveness of this set of markers in parentage tests. Consequently, the cryptic gene flow was low (0.0056) and did not bias our estimates of seed and pollen dispersal.

Among the 340 seeds of the plot, only 121 (36%) had a pollen donor within the plot, suggesting a pollen immigration rate of 64%. These 121 seeds originated from 28 pollen donors (49% of the candidate pollen donors) and a further 48 (40%) were produced through selfing, suggesting a selfing rate (*s*_offspring_) of 14.1% (48/340). Considering only outcrossing (*t*_offspring_ = 0.859), the 28 pollen donors produced an average of three seeds (ranging from 1 to 8). Furthermore, we detected 26 full-sibs and 47 half-sibs distributed among the 17 progeny-arrays. In the subplot, among the 80 seeds, 31 (39%) had a pollen donor within the plot, suggesting a pollen immigration rate of 20% (69/340), traveling distances greater than 100 m (the distance between the edges of the plot and the edges of the subplot).

Among the 147 young trees established in the subplot, 125 (85%) had at least one parent among 42 of the identified adult trees, suggesting that 15% of seeds immigrated into the subplot, traveling distances greater than 100 m (the distance between the edges of the plot and the edges of the subplot). For the assigned 125 young trees, 69 (55%) had both parents in the plot. The family structure of the 69 young trees was composed by nine (13%) self full-sibs, 51 (74%) half-sibs and nine (13%) uncategorized individuals. Eight of nine adult trees located in the subplot were the seed trees of 27 young trees, which represent only 18% of the total of young trees in the subplot.

### Pollen dispersal distance

**Figure 6:**
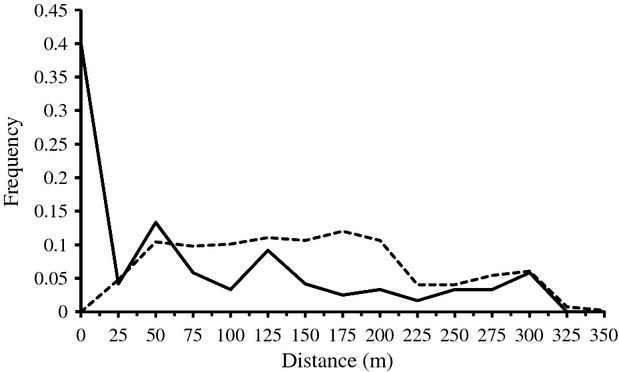
Frequency distribution of *C. langsdorffii* pollen. The black dashed line is the expected pollen dispersal distance and the black continuous line is the contemporary pollen dispersal distance.

The contemporary pollen dispersal distance ranged from 0 to 297 m, with a mean and median of 74 and 39 m, respectively (Fig. 6). The expected pollen dispersal distance ranged from 2 to 347 m, with a mean and median of 139 and 132 m, respectively (Fig. 6). There was a significant difference between the frequency distribution of contemporary pollen dispersal distance with the frequency distribution of the expected pollen dispersal distance (*D* = 0.01; *P*-value < 0.01; Fig. 6). Moreover, the average contemporary effective neighborhood pollination area (*A*_ep_) was 5.3 ha. The contemporary pollen dispersal distance that generated half-sibs (*n* = 47, mean = 134 m and median = 113 m) was significantly higher (*D* = 0.06; *P*-value = 0.02) than full-sibs (*n* = 26, mean = 103 m and median = 44 m).

### Association between spatial distance of the parents, coancestry, and DBH with the number of generated seeds

The correlation between the distance of pollen donors with the number of seeds was marginally significant (*r* = −0.253, *P-*value = 0.073), suggesting a greater tendency toward mating between spatially proximal trees than distant ones. There was a significant and positive correlation between the coancestry of the seed tree and its pollen donor with the number of seeds (*r* = 0.400, *P*-value = 0.004). Thus, the more closely related the parent pair the greater the number of seeds they generated. Furthermore, there was a significant and negative correlation between DBH of the pollen donor with the number of seeds (*r* = −0.640, *P*-value = 0.032), indicating that pollen donor trees with a higher DBH produce less seeds.

### Realized seed dispersal distance (Recruitment kernel)

**Figure 7:**
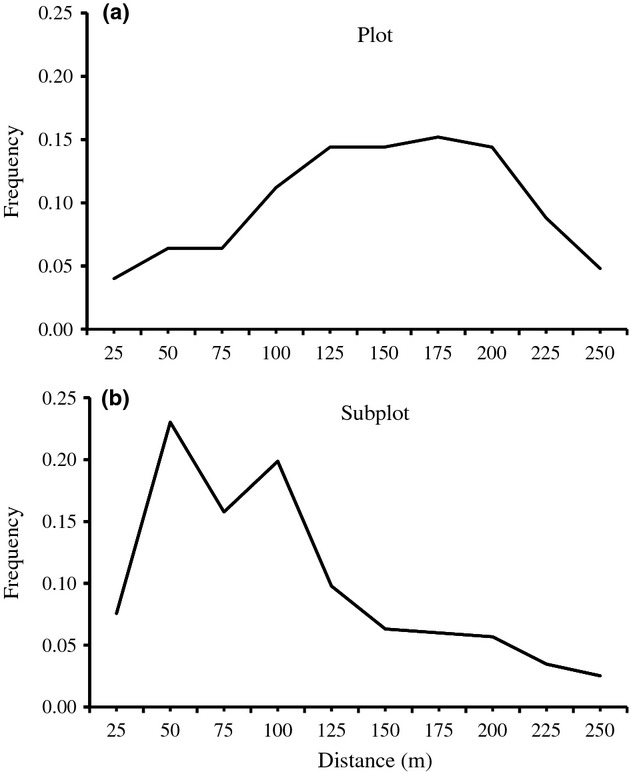
Frequency distribution of *C. langsdorffii* realized seed dispersal considering the information of assigned parents of (a) the plot and (b) only of the subplot.

In the plot, the realized seed dispersal ranged from 11 to 246 m, with a mean and median distance of 135 and 140 m, respectively (Fig. 7). The highest seed dispersal frequency distance ranged between 125 and 200 m (Fig. 7). The frequency distribution of realized seed dispersal fitted in a normal distribution function (*D* = 0.13; *P*-value = 0.16). The distance among full-sibs ranged between 7 to 115 m, with a mean distance between the closest neighbors (*R*_o_) of 70 m, and did not differ from half-sibs (range: 3.5–136 m; *R*_o_ = 48 m; *t*-test = 1.5; *P*-value = 0.13).

In the subplot, eight seed trees produced a realized seed dispersal that ranged from 11 to 124 m, with a mean and median of 59 m. The frequency distribution of realized seed dispersal also fitted in a normal distribution function (*D* = 0.09; *P*-value = 0.25).

## Discussion

Areas that changed from typical savanna to closed canopy formations are associated with an increase in forest trees species, which act to reduce fire frequency and intensity. The studies regarding this transition have concentrated their efforts in mapping the expansion and connecting it with negative global or local human disturbances (Roitman et al. 2008; Pinheiro and Durigan 2009; Tng et al. 2012; Buitenwerf et al. 2012). Our results show the positive side of closed canopy expansion, were we clearly demonstrate through genetic markers that in a 10.24 ha permanent plot of recently expanded savanna woodland, animal activities regarding pollination and seed dispersal are extremely high.

We detected a contemporary pollen immigration rate of 64% and pollen dispersal occurred up to 300 m within the plot. The contemporary effective pollination neighborhood area (*A*_ep_) was estimated to be 5.3 ha, demonstrating the foraging activity of pollen dispersers and the need to maintain savanna woodlands with areas larger than the studied plot. These results are consistent with the foraging flight distance of the most frequent pollinators to *C. langsdorffii* (*Apis mellifera*, *Scaptotrigona* cf. *depiles* and *Trigona spinipes)* that have the ability to forage effectively for up to 2 km (Pierrot and Schlindwin 2003; Araújo et al. 2004). In addition, half-sib production was 1.8 times higher than full-sibs in significant higher distances, indicating foraging activity preference for different trees at long distances. These results contrast previous research, which suggests that bee-pollination is limited to 300 m in environments with closed and dense vegetation (Dick et al. 2008; Hanson et al. 2008). Moreover, *C. langsdorffii* in this woodland savanna has higher pollen dispersal distances than the ones studied by Sebbenn et al. (2011) in the Brazilian Atlantic forest (BAF). On the other hand, due to limitations in the plots size, we observed underestimated pollen dispersal with a typical IBD pattern that was lower than the expected pollen dispersal distance (Fig. 6).

Adult trees with the largest DBH contributed less to reproduction than those with DBH closer to 25 cm, suggesting senescence of reproductive trees in the population. As the demographic structure of *C. langsdorffii* inside the plot presents itself with a reverse J-shaped diametric distribution (Fig. 3), we should expect senescence of higher diameter classes with the recruitment of younger trees. There was also a significant and positive correlation between the coancestry of the seed tree and its pollen donor with the number of seeds, because of significant inbreeding and high coancestry levels found among adult trees. In trees, mating among relative usually produces inbreeding depression (Matheson et al. 1995; Hardner and Potts 1997; Hufford and Hamrick 2003; Naito et al. 2005; Bower and Aitken 2007; Silva et al. 2011). However, this depends on the genetic load of the individuals and populations. Some studies in trees already showed that the genetic load might be different between populations (Käekkäinen et al. 1996; Hardner and Potts 1997) as well as between individuals within populations (Koelewijn et al. 1999). If two related individuals are not caring deleterious and lethal alleles, the mating between them will produce inbreeding (increase the homozygosis for identical by descent alleles within individuals), but not inbreeding depression. In our study, we found selfing rate (*s*_offspring_) of 14.1%, which is still in the limits (*s* < 0.20) of predominately outcrossing species (Goodwillie et al. 2005), suggesting that these trees may have low genetic load. Mating among relatives and selfing associated with the low *N*_e_ in adults caused significant inbreeding levels, low *H*_o_ and probably the loss of six alleles in the seeds that could lead to kin-structuring (Torimaru et al. 2007) and explain the observed SGS in young trees. Furthermore, the significant low values of *k*_R_ in seeds compared with young trees and adults, are a result of the rarefaction method that is insensitive to a large quantity of rare alleles when sample size are much higher than the minimum sample size (Leberg 2002).

Pollen generally exceeds seed dispersal (Burczyk et al. 2006; Bittencourt and Sebbenn 2007; Oddou-Muratorio and Klein 2008). In our study, pollen immigration to the subplot was 1.3 times higher than seed immigration. However, the mean and median distances of seed dispersal were much higher than pollen dispersal. First because we accounted selfing events (*d* = 0 m) in the pollen dispersal curve. Second, pollen dispersal distances are likely limited by the plot. Third, the realized seed dispersal is actually the recruitment kernel, and may be the result of several long-distance dispersal and post-dispersal survival events, with establishment of young trees. The ESA detains a high quantity and diversity of *C. langsdorffii* dispersers, it is likely that are promoting long-distance seed dispersal. Animal-generated seed shadows can affect gene movement and recruitment patterns (Jordano and Godoy 2002). Our parentage analysis revealed a hump-shaped recruitment pattern for young trees in to different scales (Fig. 7). In both cases, the minimum distance from a young tree to its parent was 11 m, with a mean of 135 and 59 for plot and subplot, respectively. Even with long-distance seed dispersal, young tree presented a clumped distribution up to 26 m (Fig. 4). Our result supports that Janzen-Connell effects influence the distribution of *C. langsdorffii* young trees in the studied plot. In this study, recruitment distance values are much higher than the distances of seed predation and mortality (20 m) reported for *C. langsdorffii* in the BAF (Pedroni 1993). In addition, spatial aggregation of tree species in tropical forests result from edaphic factors, seed dispersal, density-dependent mechanisms, and habitat specialization (Condit et al. 2000; Plotkin et al. 2000; Muller-Landau et al. 2008), and are in accordance with the JC escape hypothesis (Clark and Clark 1984; Hyatt et al. 2003). The JC escape hypothesis was also supported by other genetic study in Israel, with an expanding population of the wind-dispersed *Pinus halepensis* (Steinitz et al. 2011). The authors compared the effective seed disperse kernel, observed by the distribution of the mother-offspring distance, with the seed dispersal kernel, obtained using simulation of a mechanism of wind dispersal mode and found increased survival with distance from the mother tree.

We also suggest a lottery model (Hanski and Saccheri 2006) to explain the high distance found among full- and half-sibs in young trees. In this model, increased genetic diversity among less correlated neighbors raises the probability that a survivor genotype is present under high spatially or temporally heterogeneous environments. In our case, the spatially or temporally heterogeneous environments would be the recently expanded savanna woodland. This could explain the weak SGS found in young trees. Another non-competing explanation for our SGS in young trees is the existence of overlapping seed shadows. The realized seed dispersal distance (135 m) in *C. langsdorffii* is eight times the distance between adult trees (16.8 m) indicating overlapped seed shadows. Overlapping in the ESA plot is due to the large home range dispersers (birds and mammals) and increased tree density. These phenomena decrease correlated maternity and the relatedness among dispersed seeds (Garcia and Grivet 2011). As the adult trees existed before the savanna expansion, we cannot attribute the same mechanisms found in the young trees to explain the SGS or observed heterozygosity. What do know is that adult trees are spatially aggregated up to 120 m, show significantly higher values of observed heterozygosity (*H*_o_), lower fixation index than young trees and SGS at 40 m. The results are consistent with the recent effective population size expansion that we found, probably caused by thinning (Vieira et al. 2012), heterozygous advantage (Conte et al. 2003), and site recruitment (Tarazi et al. 2010a,b). We do believe that in this population, density-dependent effects and gene flow act together to maintain high levels of genetic diversity. Future studies should focus the contemporary evolution of savanna species, as this environment is extremely dynamic and easily altered by global and local human disturbances.

## References

[b1] Araújo ED, Costa M, Chaud-Netto J, Fowler HG (2004). Body size and flight distance in stingless bees (Hymenoptera: Meliponini): inference of flight range and possible ecological implications. Braz. J. Biol.

[b2] Ashley MV (2010). Plant parentage, pollination, and dispersal: how DNA microsatellites have altered the landscape. Crit. Rev. Plant Sci.

[b3] Bittencourt JVM, Sebbenn AM (2007). Patterns of pollen and seed dispersal in a small fragmented population of a wind pollinated *Araucaria angustifolia* in southern Brazil. Heredity.

[b4] Born C, Hardy OJ, Chevallier MH, Ossari S, Attéké C, Wickings J (2008). Small-scale spatial genetic structure in the Central African rainforest tree species *Aucoumea klaineana*: a stepwise approach to infer the impact of limited gene dispersal, population history and habitat fragmentation. Mol. Ecol.

[b5] Bower AD, Aitken SN (2007). Mating system and inbreeding depression in whitebark pine (*Pinus albicuilis* Engelm.). Tree Genet. Genomes.

[b6] Buitenwerf R, Bond WJ, Stevens N, Trollope WSW (2012). Increased tree densities in South African savannas: > 50 years of data suggests CO2 as a driver. Glob. Change Biol.

[b7] Burczyk J, Adams WT, Birkes DS, Chybicki IJ (2006). Using genetic markers to directly estimate gene flow and reproductive success parameters in plants on the basis of naturally regenerated seedlings. Genetics.

[b8] Carvalho PER (2003). Espécies Florestais Brasileiras: recomendações silviculturais, potencialidades e uso da madeira.

[b10] Cavers S, Degen B, Caron H, Lemes MR, Margis R, Salgueiro F (2005). Optimal sampling strategy for estimation of spatial genetic structure in tree populations. Heredity.

[b11] Choo J, Juenger TE, Simpson BB (2012). Consequences of frugivore-mediated seed dispersal for the spatial and genetic structures of a neotropical palm. Mol. Ecol.

[b12] Chung MI, Nason JD, Chung MG (2011). Significant demographic and fine-scale genetic structure in expanding and senescing populations of the terrestrial orchid *Cymbidium goeringii* (Orchidaceae). Am. J. Bot.

[b13] Ciampi AY, Brondani RPV, Grattapaglia D (2000). Desenvolvimento de marcadores microssatélites para *Copaifera langsdorffii* Desf. (copaíba) Leguminosae – Caesalpinoideae e otimização de sistemas fluorescentes de genotipagem multiloco. Bol. Pesq. Embrapa Recur. Gen. Biotec.

[b14] Clark DA, Clark DB (1984). Spacing dynamics of a tropical rainforest tree: evaluation of the Janzen-Connell model. Am. Nat.

[b15] Cockerham CC (1969). Variance of gene frequency. Evolution.

[b16] Condit R, Ashton PS, Baker P, Bunyavejchewin S, Gunatilleke S, Gunatilleke N (2000). Spatial patterns in the distribution of tropical tree species. Science.

[b17] Connell JH, Boer PJ, Gradwell GR (1971). On the role of natural enemies in preventing competitive exclusion in some marine animals and rain forest trees. Dynamics of populations.

[b18] Conte R, Nodari RO, Vencovsky R, Reis MS (2003). Genetic diversity and recruitment of the tropical palm, *Euterpe edulis* Mart., in a natural population from the Brazilian Atlantic Forest. Heredity.

[b19] Di Rienzo A, Peterson AC, Garza JC, Valdes AM, Slatkin M, Freimer NB (1994). Mutational processes of simple-sequence repeat loci in human populations. Proc. Natl Acad. Sci. USA.

[b20] Dick CW, Hardy OJ, Jones FA, Petit RJ (2008). Spatial scales of pollen and seed-mediated gene flow in tropical rain forest trees. Trop. Plant Biol.

[b21] Dow BD, Ashley MV (1996). High levels of gene flow in Bur Oak revealed by paternity analysis using microsatellites. J. Hered.

[b22] Doyle JJ, Doyle JL (1990). Isolation of plant DNA from fresh tissue. Focus.

[b24] Epperson BK (2003). Geographical genetics.

[b25] Felfini JM, Resende AV, Silva Júnior MC, Silva MA (2000). Changes in the floristic composition of cerrado *sensu stricto* in Brazil over a 9-year period. J. Trop. Ecol.

[b26] Freitas CV, Oliveira PE (2002). Biologia reprodutiva de *Copaifera langsdorffii* Desf. (Leguminosae, Caesalpinioideae). Rev. Bras. Bot.

[b27] Garcia C, Grivet D (2011). Molecular insights into seed dispersal mutualisms driving plant population recruitment. Acta Oecol.

[b28] Goodwillie C, Kalisz S, Eckert CG (2005). The evolutionary enigma of mixed mating systems in plants: occurrence, theoretical explanations, and empirical evidence. Annu. Rev. Ecol. Evol. Syst.

[b29] Goudet J (1995). FSTAT. (Version 2.9.3.2.): a computer program to calculate *F*-statistics. J. Hered.

[b30] Hamrick JL, Murawski DA, Nason JD (1993). The influence of seed dispersal mechanisms on the genetic structure of tropical tree populations. Vegetatio.

[b31] Hanski I, Saccheri I (2006). Molecular-level variation affects population growth in a butterfly metapopulation. PLoS Biol.

[b32] Hanson TR, Brunsfeld SJ, Finegan B, Waits LP (2008). Pollen dispersal and genetic structure of the tropical tree *Dipteryx panamensis* in a fragmented Costa Rican landscape. Mol. Ecol.

[b33] Hardesty BD, Hubbell S, Bermingham E (2006). Genetic evidence of frequent long distance recruitment in a vertebrate-dispersed tree. Ecol. Lett.

[b34] Hardner CM, Potts BM (1997). Postdispersal selection following mixed mating in *Eucalyptus regnans*. Evolution.

[b35] Hardy OJ, Vekemans X (2002). SPAGeDI: a versatile computer program to analyse spatial genetic structure at the individual or population levels. Mol. Ecol. Notes.

[b37] Hardy OJ, Maggia L, Bandou E, Breyne P, Caron H, Chevallier MH (2006). Fine-scale genetic structure and gene dispersal inferences in 10 Neotropical tree species. Mol. Ecol.

[b38] Henriques RPD, Hay JD, Oliveira OS, Marquis RJ (2002). Pattern and dynamics of plant population. The cerrados of Brazil: ecology and natural history of a neotropical savanna.

[b39] Higgins SI, Bond WJ, February EC, Bronn A, Euston-Brown DIW, Enslin B (2007). Effects of four decades of fire manipulation on woody vegetation structure in savanna. Ecology.

[b40] Hoffman JI, Amos W (2005). Microsatellite genotyping errors: detection approaches, common sources and consequences for paternal exclusion. Mol. Ecol.

[b41] Hufford KM, Hamrick JL (2003). Viability selection at three early life stages of the tropical tree, *Platypodium elegans* (Fabaceae, Papilionoideae). Evolution.

[b42] Hyatt LA, Rosenberg MS, Howard TG, Bole G, Fang W, Anastasia J (2003). The distance dependence prediction of the Janzen-Connell hypothesis: a meta-analysis. Oikos.

[b43] Janzen DH (1970). Herbivores and the number of tree species in tropical forests. Am. Nat.

[b44] Jordano P, Godoy JA, Levey DJ, Silva W, Galetti M (2002). Frugivore-generated seed shadows: a landscape view of demographic and genetic effects. Frugivores and seed dispersal: ecological, evolutionary, and conservation perspectives.

[b45] Käekkäinen K, Koski V, Savolainen O (1996). Geographical variation in the inbreeding depression of scots pine. Evolution.

[b46] Kalinowski ST, Taper ML, Marshall TC (2007). Revising how the computer program CERVUS accommodates genotyping error increases success in paternity assignment. Mol. Ecol.

[b48] Klink CA, Machado R (2005). Conservation of the Brazilian Cerrado. Conserv. Biol.

[b49] Koelewijn HP, Koski V, Savolainen O (1999). Magnitude and timing of inbreeding depression in Scots Pine (*Pinus sylvestris* L.). Evolution.

[b51] Leberg PL (2002). Estimating allelic richness: effects of sample size and bottlenecks. Mol. Ecol.

[b52] Levin DA (1988). Consequences of stochastic elements in plant migration. Am. Nat.

[b53] Loiselle BA, Sork VL, Nason J, Graham C (1995). Spatial genetic structure of a tropical understory shrub, *Psychotria officinalis* (Rubiaceae). Am. J. Bot.

[b54] Luikart G, Cornuet JM (1998). Empirical evaluation of a test for identifying recently bottlenecked populations from allele frequency data. Conserv. Biol.

[b55] Marshall TC, Slate J, Kruuk LEB, Pemberton JM (1998). Statistical confidence for likelihood-based paternity inference in natural populations. Mol. Ecol.

[b56] Matheson AC, White TL, Powel GR (1995). Effects of inbreeding on growth, stem form and rust resistence in *Pinus elliottii*. Silvae Genet.

[b57] Meagher TR (1986). Analysis of paternity within a natural population of *Chamaelirium luteum*. I. Identification of most-likely male parents. Am. Nat.

[b58] Meagher TR, Thompson E (1986). The relationship between single parent and parent pair genetic likelihoods in genealogy reconstruction. Theor. Popul. Biol.

[b59] Moreira AG (2000). Effects of fire protection on savanna structure in Central Brazil. J. Biogeogr.

[b60] Muller-Landau HC, Wright SJ, Calderón O, Condit R, Hubbell SP (2008). Interspecific variation in primary seed dispersal in a tropical forest. J. Ecol.

[b61] Naito Y, Konuma A, Iwata YS, Seiwa K, Okuda T, Lee SL (2005). Selfing and inbreeding depression in seeds and seedlings of *Neobalanocarpus heimii* (Dipterocarpaceae). J. Plant Resour.

[b62] Nathan R (2006). Long-distance dispersal of plants. Science.

[b63] Nathan R, Casagrandi R (2004). A simple mechanistic model of seed dispersal, predation and plant establishment: Janzen-Connell and beyond. J. Ecol.

[b64] Oddou-Muratorio S, Klein EK (2008). Comparing direct vs. indirect estimates of gene flow within a population of a scattered tree species. Mol. Ecol.

[b65] Ohta T, Kimura M (1973). A model of mutation appropriate to estimate the number of electrophoretically detectable alleles in a finite population. Genet. Res.

[b66] Oosterhout CV, Hutchinson WF, Wills DPM, Shipley P (2004). Micro-checker: software for identifying and correcting genotyping errors in microsatellite data. Mol. Ecol. Notes.

[b67] Pedroni F (1993). Dispersão e recrutamento de Copaifera langsdorffii Desf. (Caesalpiniaceae) na reserva municipal de Santa Genebra.

[b68] Pierrot LM, Schlindwin C (2003). Variation in daily flight activity and foraging patterns in colonies of uruçu – *Melipona scutellaris* Latreille (Apidae, Meliponini). Rev. Bras. Zool.

[b69] Pinheiro ES, Durigan G (2009). Spatial and temporal dynamics (1962–2006) of Cerrado vegetation types in a protected area, southeastern Brazil. Rev. Bras. Bot.

[b70] Piry S, Luikart G, Corneut JJ (1999). BOTTLENECK: a computer program for detecting recent reductions in the effective population size using allele frequency data. J. Hered.

[b71] Plotkin JB, Potts MD, Lesliea N, Manokaran N, Lafrankieb J, Ashton PS (2000). Species-area curves, spatial aggregation, and habitat specialization in tropical forests. J. Theor. Biol.

[b72] Puyravaud JP, Pascal JP, Dufour C (1994). Ecotone structure as an indicator of changing forest-Savanna boundaries (Linganamakki Region, Southern India). J. Biogeogr.

[b73] Ribeiro MC, Metzger JP, Martensen AC, Ponzoni FJ, Hirota MM (2009). The Brazilian Atlantic Forest: how much is left, and how is the remaining forest distributed? Implications for conservation. Biol. Conserv.

[b74] Ritland K (2002). Extensions of models for the estimation of mating systems using n independent loci. Heredity.

[b75] Robledo-Arnuncio JJ, Austerlitz F (2006). Pollen dispersal in spatially aggregated populations. Am. Nat.

[b76] Roitman I, Felfili JM, Rezende AV (2008). Tree dynamics of a fire-protected cerrado *sensu stricto* surrounded by forest plantations, over a 13-year period (1991–2004) in Bahia, Brazil. Plant Ecol.

[b77] Rousset F (2000). Genetic differentiation between individuals. J. Evol. Biol.

[b78] Russell-Smith J, Stanton PJ, Edwards AC, Whitehead PJ (2004). Rain forest invasion of eucalypt-dominated woodland savanna, Iron Range, North-eastern Australia: II. Rates of landscape change. J. Biogeogr.

[b79] Sebbenn AM, Carvalho ACM, Freitas MLM, Moraes SMB, Gaino APSC, Silva JM (2011). Low levels of realized seed and pollen gene flow and strong spatial genetic structure in a small, isolated and fragmented population of the tropical tree *Copaifera langsdorffii* Desf. Heredity.

[b80] Silva JC, Hardner C, Tilyard P, Potts BM (2011). The effects of age and environment on the expression of inbreeding depression in *Eucalyptus globulus*. Heredity.

[b81] Sokal RR, Rohlf FJ (1989). Biometry: principles and practices of statistics in biological research.

[b82] Steinitz O, Troupin D, Vendramin GG, Nathan R (2011). Genetic evidence for a Janzen-Connell recruitment pattern in reproductive offspring of *Pinus halepensis* trees. Mol. Ecol.

[b83] Tarazi R, Mantovani A, Reis MS (2010a). Fine-scale spatial genetic structure and allozymic diversity in natural populations of *Ocotea catharinensis* Mez. (Lauraceae). Conserv. Genet.

[b84] Tarazi R, Moreno MA, Gandara FB, Ferraz EM, Moraes MLT, Vinson CC (2010b). High levels of genetic differentiation and selfing in the Brazilian cerrado fruit tree *Dipteryx alata* Vog. (Fabaceae). Genet. Mol. Biol.

[b85] Tarazi R, Sebbenn AM, Mollinari M, Vencovsky R (2010c). Mendelian inheritance, linkage and linkage disequilibrium in microsatellite loci of *Copaifera langsdorffii* Desf. Conserv. Genet. Resour.

[b86] Tng DYP, Murphy BP, Weber E, Sanders G, Williamson GJ, Kemp J (2012). Humid tropical rain forest has expanded into eucalypt forest and savanna over the last 50 years. Ecol. Evol.

[b87] Torimaru T, Tani N, Tsumura Y, Nishimura N, Tomaru N (2007). Effects of kin-structured seed dispersal on the genetic structure of the clonal dioecious shrub *Ilex leucoclada*. Evolution.

[b88] Troupin D, Nathan R, Vendramin GG (2006). Analysis of spatial genetic structure in an expanding *Pinus halepensis* population reveals development of fine-scale genetic clustering over time. Mol. Ecol.

[b89] Vekemans X, Hardy OJ (2004). New insights from fine-scale spatial genetic structure analyses in plant populations. Mol. Ecol.

[b90] Vieira FA, Fajardo CG, Souza AM, Reis CAF, Carvalho D (2012). Fine-scale genetic dynamics of a dominant neotropical tree in the threatened Brazilian Atlantic Rainforest. Tree Genet. Genomes.

[b91] Ward M, Dick CW, Gribel R, Lowe AJ (2005). To self, or not to self. A review of outcrossing and pollen-mediated gene flow in neotropical trees. Heredity.

[b92] Wiegand T (2003). http://www.thorsten-wiegand.de/towi_programita.html.

[b93] Wiegand T, Moloney KA (2004). Rings, circles, and null-models for point pattern analysis in ecology. Oikos.

